# Hematology and plasma biochemistry of wild-caught Indian cobra *Naja naja* (Linnaeus, 1758)

**DOI:** 10.1186/1678-9199-20-14

**Published:** 2014-04-15

**Authors:** Siba Prasad Parida, Sushil Kumar Dutta, Arttatrana Pal

**Affiliations:** 1 Department of Zoology, Utkal University, Vani Vihar, Bhubaneswar, India; 2 School of Biotechnology, KIIT University, Bhubaneswar, Odisha 751024, India

**Keywords:** Reference range, Snake, *Naja naja*, Hematology, Plasma biochemistry

## Abstract

**Background:**

Hematology and plasma biochemistry parameters are useful in the assessment and management of snake physiological status. Although reference ranges are readily available for many snake species, they are lacking for most venomous ophidians. We determined hematology and plasma biochemistry reference ranges for the wild-caught Indian cobra, *Naja naja.*

**Results:**

Blood samples, taken from the ventral tail vein, were assessed for erythrocyte count, total leukocyte count, hemoglobin concentration, hematocrit, mean corpuscular volume, mean corpuscular hemoglobin, and mean corpuscular hemoglobin concentration, considering the sex of snakes.

Results revealed the erythrocyte numbers (male, 390000 ± 12503.33/mm^3^ and female, 347500 ± 7505.55/mm^3^), shapes and the centrally located oval nuclei. Leukocytes were round, circular or disk-shaped, and the mean size was larger in male than female snakes. The maximum number of leukocytes was found to be 11700 ± 100/mm^3^ in male and 12100 ± 200/mm^3^ in female snakes, and mean values of differential leukocyte count differed statistically between male and female snakes. The total leukocyte levels were found to be higher in female snakes, but the levels of hemoglobin, hematocrit, and MCV values were higher in male snakes. However, the MCH and MCHC values remained higher in female snakes throughout the study period. Mean protein and cholesterol contents differed significantly between male (45.32 ± 1.76 and 3.76 ± 0.06 mg/mL) and female (12.47 ± 0.82 and 4.72 ± 0.2 mg/mL) snakes.

**Conclusions:**

In conclusion, monitoring snake hematological and biochemical parameters can serve as a means to evaluate the physiological and health status of *N. naja* populations, which may be a useful indicator of their environmental status.

## Background

Hematology and plasma biochemistry values are useful in assessing the health and fitness of reptiles kept in captivity for research, as pets, or in zoos [1-3]. The combination of many parameters required for detection of physiological stress and clinical evaluation in reptiles such as the complete blood count can identify dehydration, anemia, inflammatory diseases, parasitemia, hematopoietic disorders, and hemostatic alterations [4,5]. In addition, blood profiles provide a minimally invasive tool to assess the health condition in live reptiles that may be influenced by a number of internal and environmental factors [6]. However, hematological parameters and plasma chemistry of reptiles show fluctuations depending on seasonality and motor activity, phase of reproductive cycle, age, sex, geophysical conditions of the habitat, acclimation temperature and photoperiod [7]. These parameters can vary through the annual cycle or even throughout the life of the individuals [8-10]. Many studies have reported on different biochemical and hematological parameters of reptiles including a few snake species as a baseline in order to correlate their physiology and evolution [11-15]. However, a thorough knowledge of snake physiology is becoming increasingly imperative due to diagnostic demand, economic importance, the need for conservation actions and their role as pets. So far, few studies have been published in the literature on the hematology and plasma biochemistry reference ranges for non-venomous snakes, while venomous snake species have been studied very sparsely [5,16-21].

Recently, the majority of the hematological studies carried out on different reptile species have dealt with blood composition, as well as blood cell counts and sizes [15,16]. However, the hematological studies on various snake species are related only to blood cell sizes and counts [22,23]. Most of the earlier works on reptiles are confined to European species and describe the cell composition and the chemistry of the blood [24]. The circulating blood cells of different reptiles have been described by multiple authors [25,26]. Campbell [27] reported that total erythrocyte and leukocyte counts in reptiles fail to account for the nuances and the fact that all the cells in the peripheral blood are nucleated. Hematology of green iguanas has been a subject of interest for a number of authors. The results of trials showed a significant degree of variation due to different animal selection methods and technical differences in blood sample treatment [28-30]. Apart from mature blood cells, peripheral blood film of healthy reptiles contains immature elements [28]. So far, there are still no referential data on detailed blood biochemistry levels in venomous snakes. Evidence for the presence of albumin-like protein in reptiles is limited; 3% to 7% of blood plasma in reptiles is comprised of a complex mixture of proteins [8]. The cholesterol concentration in reptile blood varies among species, as reported in *Alligator mississippiensis* (50 mg), *Crotalus atrox* (100–172 mg), *Vipera aspis* (220 mg), and *Sitana ponticeriana* (4–13 mg); [8,31-33].

The distribution of the Indian cobra – a highly poisonous snake that lives in plains, jungles, open fields and areas heavily populated by humans – ranges from sea-level up to 6,600 feet [34]. This species normally feeds on rodents, toads, frogs, birds and other snakes in areas inhabited by humans including farms and outskirts of urban areas. Many publications have reported different aspects of the Indian cobra, but there is no information on the hematology and plasma chemistry of specimens of this species living in India. Therefore, the present study aimed to determine the selective hematological parameters and plasma chemistry of Indian cobra specimens at Bhubaneswar, India. These findings will serve as baseline reference data for future health assessment studies of the Indian cobra, as well as for the epidemiologic, conservation and captive-breeding studies, among other applications.

## Methods

### Animals

Twenty-seven adult males and thirty-two adult females snakes were collected from various localities in the city of Bhubaneswar (20°18′05.40′′ N and 85°50′28.29′′ E), India, from 2003 to 2011. Before blood collection, specimens were weighed and their total length was measured. The males presented weights from 295 to 320 g (304 ± 9 g) whereas females weighed 275 to 295 g (282.5 ± 13.5 g). Male snake body lengths ranged from 97 to 120 cm (108.5 ± 11.5 cm) versus 98 to 112 cm (105 ± 7) among females.

### Blood collection

Blood was collected from each snake’s ventral tail vain by immobilizing the head and cranial half of the body, as described by Tosunoglu *et al.*[35]. Briefly, the venipuncture site was cleaned and aseptically prepared prior to blood collection. A needle (22- to 25-gauge) was inserted at an angle of 45-60° between the scales on ventral midline and, once blood appeared in the needle hub, held steady while continuing to apply gentle negative pressure. Later, the snakes were released back to the closest forest areas. Whole blood smears were obtained using a push slide technique, air-dried, fixed with methanol and stained with Wright’s-Giemsa [10]. Five blood smears were prepared per individual. The proportions of heterophils including potential eosinophils, which could not be definitively identified by morphology alone, lymphocytes, basophils, and combined monocytes were classified through manual counts of blood smears as previously published by us [9,10]. The rest of the blood was centrifuged, and the plasma was separated for biochemical analysis.

### Hematological, plasma protein and cholesterol analysis

The hemoglobin (Hb) concentration was estimated by a Sahlis hemometer and expressed in g% [10]. For counting the number of red blood cells (RBCs) and white blood cells (WBCs), we followed standard procedure as published earlier by us and also by others [9,35]. The results were expressed as the number of RBCs or WBCs per 1 mm^3^ of blood. Briefly, the RBCs and WBCs were quantified using a Neubauer hemocytometer, with dilution being performed by standard Hayem’s solution for RBCs and Turk’s solution for WBCs. Hematocrit (HCT) was determined using the microhematocrit method [9]. The tubes were then spun in a microhematocrit centrifuge for five minutes at 12,000 rpm and the HCT was calculated with the total blood level divided by the blood cell level. The mean corpuscular volume (MCV), mean corpuscular hemoglobin (MCH), and mean corpuscular hemoglobin concentrations (MCHC) were calculated, taking the above results into consideration [8,9]. For the differential leukocyte count (DLC), the blood smears were fixed with methanol and stained with Wright’s-Giemsa and then examined under a microscope. The percentages of different leukocytes were determined after counting a total of 100 cells. Sizes of RBCs, WBCs, and nuclei were measured by an ocular micrometer (ERMA, Japan). The sizes of RBCs and their nuclei were obtained by measuring their long and short axes (length and breadth) in the case of elliptical RBCs. For round RBCs, only the diameter was considered. The areas of RBCs and WBCs were calculated according to the following formula: elliptical RBC = short axis × long axis × 0.7854 and round RBC/WBC = πr^2^[10].

For the measurement of blood protein and cholesterol levels of snakes, we followed standard procedure, as published earlier by us, in other reptiles [8-10]. Protein content of the plasma was determined according to the method of Lowry *et al.*[36]. Absorbency of the color found was measured against an appropriate blank. The protein content was expressed as mg/mL of plasma. Cholesterol content of the plasma was estimated according to the method of Rosenthal *et al.*[37]. Absorbency was assessed against an appropriate blank, and cholesterol content was expressed as mg/mL of plasma.

### Statistical analysis

Hematological and plasma biochemistry data resulting from our study were transformed into means and standard deviation (SD) via the software SPSS 17 for Windows. Significant differences between means were determined using an independent sample *t* test model. Results were considered significant at p < 0.05.

## Results

**Figure 1:**
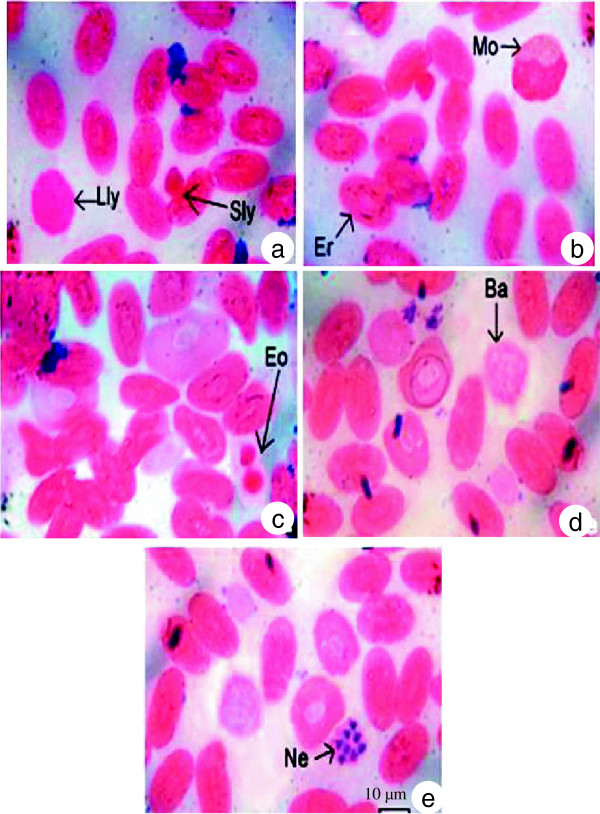
**Morphology and staining characteristic of (a) large lymphocytes (Lly), small lymphocytes (Sly), (b) erythrocytes (Er), normocytes (Mo), (c) eosinophils, (d) basophils and (e) heterophils (Ne) of the circulating blood of *Naja naja.***

Morphologically, WBCs and RBCs of *N. naja* were observed to be oval or elliptical in shape with an oval nucleus (Figure 1). Sometimes round RBCs with a round nucleus were observed. In Giemsa stain, the RBC cytoplasm presented a light violet color whereas in some slides, the nucleus of RBCs was deep pink in color. As shown in Table 1, in male snakes the long (18.08 ± 0.20 μm) and short (10.87 ± 0.82 μm) axes of erythrocytes remained equal in all cells. Similarly, in female snakes the long (17.63 ± 0.67 μm) and short (11.17 ± 0.31 μm) erythrocyte axes remained equal in size in all cells. The area occupied by the RBCs in female snakes (140.73 ± 4.02 μm^2^) remained less than that in male ones (146.46 ± 9.58 μm^2^). Although the length and breadth of nuclei in males remained identical in all cells, it varied in female snakes. The nuclear area remained identical in all the cells in male and female snakes. The ratio between cells and nuclei averaged 4.34 ± 0.69 in male snakes versus 5.76 ± 0.94 in females.

**Table 1:** **Average length and breadth of the erythrocytes (LB), nuclei (L′B′), surface area of erythrocyte (S) and nuclei (S′) in male (n = 27) and female (n = 32) *Naja naja *** **snakes (p < 0.05)**

**Parameter**	**Male (n = 27)**		**Female (n = 32)**	
	**Range**	**Mean ± SD**	**Range**	**Mean ± SD**
L (μm)	17.94-18.23	18.085 ± 0.20	16.26-18.08	17.63 ± 0.67
B (μm)	10.18-11.72	10.87 ± 0.82	10.95-11.4	11.17 ± 0.31
S (μm^2^)	132.24-172.81	146.46 ± 9.58	137.89-143.58	140.73 ± 4.02
L′ (μm)	8.28-8.76	8.52 ± 0.339	6.72-8.54	7.86 ± 0.89
B′ (μm)	5.52-5.84	5.68 ± 0.22	4.14-4.67	4.40 ± 0.37
S′ (μm^2^)	35.89-38.26	37.07 ± 1.67	22.65-27.23	25.78 ± 0.47

The observed WBCs could be classified into the following five types: lymphocytes, monocytes, eosinophils, basophils and heterophils, the first two being agranulocytes while the rest were granulocytes. Lymphocytes were round or spherical in shape (Figure 1a). According to size, they were categorized as large and small lymphocytes. The nuclei were round in both large and small lymphocytes and occupied almost the entire cell. As shown in Table 2, male snakes presented larger large lymphocytes (18.93 ± 0.86 μm) than their female counterparts (16.36 ± 0.89 μm) snakes. Similarly, small lymphocytes were larger in male snakes (7.65 ± 1.04 μm) than in females (5.28 to 7.06 μm). Monocytes presented an oval or round shape with an eccentric nucleus (Figure 1b). Some nuclei were kidney shaped while others were elongated. The nucleus was darkly stained while the cytoplasm was lighter. The size of monocytes with an average diameter of 12.87 ± 1.18 μm in male snakes versus 11.72 ± 1.08 μm in females. Eosinophils were identified by their granular appearance (Figure 1c). The granules were darkly stained. The nucleus was either two-lobed or concentrated at one end of the cell, appearing to be thick and slightly notched in the middle. The cytoplasm was lightly stained. The diameter of eosinophils averaged 9.32 ± 1.02 μm in male snakes versus 7.98 ± 1.29 μm in females. Basophils, identified by the presence of granules over the entire cell including the nucleus (Figure 1d), were oval-shaped with a lobed nucleus. Although the basophil diameters were identical in female snakes (5.64 μm), they presented some variation in males, averaging 4.09 ± 1.24 μm. Heterophils were circular with four to five lobes in the nucleus (Figure 1). The nuclei appeared dark pink while the cytoplasm remained lighter in color. The heterophil diameters averaged 5.21 ± 1.13 μm in male snakes versus 6.76 ± 1.28 μm among females.

**Table 2:** **Sizes of white blood cells (WBCs) and differential leukocyte counts (DLCs) in male (n = 27) and female (n = 32) *Naja naja *** **snakes (p < 0.05)**

**Cell type**	**Male (n = 27)**				**Female (n = 32)**			
	**WBC size (μm)**		**DLC count (%)**		**WBC size (μm)**		**DLC count (%)**	
	**Range**	**Mean ± SD**	**Range**	**Mean ± SD**	**Range**	**Mean ± SD**	**Range**	**Mean ± SD**
Large lymphocytes	18.16-20.05	18.93 ± 0.86	42-55	49 ± 5.43	15.47-16.94	16.36 ± 0.89	40-52	47.26 ± 4.4
Small lymphocytes	7.06-8.57	7.65 ± 1.04			5.28-7.06	6.76 ± 0.89		
Monocytes	12.98-15.34	12.87 ± 1.18	2-6	4 ± 2	10.96-12.56	11.72 ± 1.08	1-4	2.66 ± 1.52.
Eosinophils	8.78-9.88	9.32 ± 1.02	2-5	3.66 ± 1.52	6.86-8.43	7.98 ± 1.29	1-4	2.66 ± 1.52
Basophils	2.66-5.75	4.09 ± 1.24	3-6	4.66 ± 1.52	5.64		3-7	5 ± 2
Heterophils	4.35-5.73	5.21 ± 1.13	22-29	25.33 ± 3.51	5.82-7.15	6.76 ± 1.28	19-28	23.66 ± 4.5

As shown in Table 3, hemoglobin content ranged from 6.5 to 7.2 g/100 mL in male snakes and 6.2 to 6.8 g/100 mL in females. The PCV value ranged from 26.8 to 32.7% in males whereas among female snakes it varied between 21.6 and 28.2%, which indicates that PCV remained higher in males throughout the study period. The MCV value for male snakes ranged between 304.1 and 439.9 fL and 289.7 and 389.5 fL among females. There was a significant difference in MCH value among male and female snakes, 103.7 to 125.3 pg in males and 136.1 to 157.2 pg in females. The MCHC value ranged from 36.5 to 41.8% in males, whereas for female snakes it varied between 39.4% and 45.2%.

**Table 3:** **The numbers of RBC and WBC, Hb contents, PCV, MCV, MCH, MCHC, amount of protein and cholesterol in the blood in male (n = 27) and female (n = 32) snakes of *Naja naja *** **(p < 0.05)**

**Parameter**	**Male (n = 27)**		**Female (n = 32)**	
	**Range**	**Mean ± SD**	**Range**	**Mean ± SD**
RBC number (mm^3^)	380000-400000	390000 ± 12503.33	340000-355000	347500 ± 7505.55
WBC number (mm^3^)	11800-11600	11700 ± 100	11900-12300	12100 ± 200
Hb (g%)	6.5 to 7.2	6.9 ± 0.1	6.2-6.8	6.5 ± 0.2
PCV (%)	26.8-32.7	29.8 ± 4.2	21.6-28.2	25.4 ± 3.9
MCV (fL)	304.1-439.9	386.7 ± 63.8	289.7-389.5	348.2 ± 59.33
MCH (pg)	103.7-125.3	118.6 ± 23.1	136.1-157.2	142.4 ± 19.5
MCHC (%)	36.5 to 41.8	38.8 ± 1.9	39.4-45.2	42.6 ± 1.4
Proteins (mg/mL of serum)	41.83-47.56	45.32 ± 1.76	12.25-13.23	12.47 ± 0.82
Cholesterol (mg/mL of serum)	3.68-3.89	3.76 ± 0.06	4.53 to 4.92	4.72 ± 0.2

As shown in Table 3, the total number of RBCs in males ranged from 380000 to 400000/mm^3^ versus 340000 to 355000/mm^3^ in females. Conversely, the WBC range in males, from 11800 to 11600/mm^3^, was below that of females, 11900 to 12300/mm^3^. Differential leucocyte counts are displayed in Table 3. The number of large and small lymphocytes varied from 42 to 55% in male snakes compared with 40 to 52% among females. The percentage of monocytes ranged from 2 to 6 in males but 1 to 4 in females. The percentage of eosinophils varied from 2 to 5 in males versus 1 to 4 in female snakes. The basophil percentages were similar in males and females, from 3 to 6 and 3 to 7, respectively. Heterophils ranged from 22 to 29 percent in male snakes compared with 19 to 28 in females. As shown in Table 3, the protein content was much higher in males – from 41.83 to 47.56 mg/mL of blood serum – than females, which presented 12.75 to 13.23 mg/mL. Female snakes showed higher cholesterol content – from 4.53 to 4.92 mg/mL of blood serum– than the male range of 3.68 to 3.89 mg/mL.

## Discussion

Hematological and plasma biochemistry data are needed to characterize the health status of snake populations over time and to relate health to habitat quality. A profile that combines hematological and biochemical aspects is required for diagnosis and also for the treatment of diseases in veterinary medicine [5]. However, these measurements may vary depending on various external as well as physiological factors such as sex, age, pregnancy, physical exercise, weather, stress, altitude, and captivity [25,38]. The essential task of identifying the causes of disease in snakes presents a challenge for the veterinary practitioner. Despite the existence of several excellent references used as guides, the relevant scientific literature is far from comprehensive on specific conditions in the unusual reptile patient including snakes and the incisive expert needs to employ all available diagnostic skills to manage these cases. The use of clinical pathology greatly enhances this endeavor. In addition, it is important to establish reference ranges of hematological and biochemical parameters for disease diagnosis, health monitoring, and in the detection of any ecological and geographical differences among the reptile species including snakes [5,35].

Several publications reported that the components and biochemical composition of blood vary with season, age, molting, pathological conditions, reproductive state, and ecological factors in different poikilothermic animals [7,9]. Reptiles are a heterogeneous group of vertebrates with respect to their blood cell morphology. The differential blood cell count is important in determining the health condition of animals. Lizards generally have higher erythrocyte counts than snakes, but turtles have the lowest. Many researchers have stated that there is great intra- and interspecific variations of blood cell count in snakes [5,23,24]. As to erythrocyte measurements among snakes, many researchers have reported homogeneity, except for *Typhlops vermicularis*. The erythrocytes of *T. vermicularis* were found to differ by being smaller and more elongated [25]. The blood of *T. vermicularis* contains the same erythrocyte count as other colubrid species, but in a smaller volume, which places the species among the most specialized snakes in this respect. Among the experimental animal in the present study, *N. naja* differs in its length, weight and month of collection. The results obtained were similar to the reference ranges for other snakes.

The shape and size of RBCs are variable for different vertebrates and morphologically similar among various species of reptiles [25]. The morphology of RBCs was stated to have an oval shape in other ectothermic vertebrates. The semivenomous snake species *Malpolon monspessulanus* and *Telescopus fallax* were found to have the lowest RBC, Hb, and HCT values in comparison with those of other colubrid species [31]. According to Tok *et al.*[22], the shortest RBC belongs to the species *M. monspessulanus*. Nevertheless, clinical results similar to other species were obtained in *M. monspessulanus*. In the present study, our finding in *N. naja* coincides with those of others. Erythrocyte nuclei become condensed, stain darker as the cells age, and are centrally located. The identification of the cells in a smear obtained from circulating snake blood is often difficult since these animals present cells of all ages and show intermediate stages between the different cell types.

Inter- and even intra-family differences in cells are such that, with present knowledge, it is virtually impossible to determine morphological characteristics for each cell type. Only those of erythrocytes are becoming well known. As transitional forms of cells are very common, it is often difficult to identify different cells in a smear. The shape and size of RBCs are oval or elongated or elliptical in the case of snakes and the present study reported oval or elliptical shape in *N. naja.* Sometimes round RBCs and a round nucleus are present in *N. naja*. The size of RBCs is highly similar in the male and female snakes of this species. The shape of different leukocytes is almost round, circular, or disk-shaped. However, a more accurate assessment of disease in a reptile patient may be obtained by observing the abnormal morphology of the leukocytes [39]. The literature enumerated 1.24 × 10^6^ and 1.8 × 10^6^ RBCs in different reptiles. Herein our findings in *N. naja* coincide with the results reported earlier in other reptiles [8,9]. The number of circulating eosinophils in normal reptiles varies with species and season changes, i.e., in the winter during hibernation the eosinophil counts are usually highest. Our findings of RBC and WBC counts of this snake closely coincide with those reported in other reptiles including snakes [5,8,40]. On the other hand, comparing the hematological values of the venomous snake species *N. naja* produced highly similar results.

When the data from two semivenomous snake species, *M. monspessulanus* and *T. fallax*, were compared with our studies on the venomous snake *N. naja*, the erythrocyte count was found to be higher; the leukocyte count, Hb, MCH, and MCHC values all lower; and the HCT and MCV values similar in comparison with species in the family Crotalidae, namely *Bothrops jararacussu*[31] and *Bothrops ammodytoides*[26]. As to the clinical values of a few snake species, Tosunoglu *et al.*[35] found a lower MCHC value in *T. vermicularis* than in *T. fallax.* In addition, Vasaruchapong *et al.*[5] demonstrated in *Naja kaouthia*, that the hematological values of packed cell volume, total red blood cell count, MCV, and total white blood cell count did not differ significantly among species.

Regarding hemoglobin content, Szarski and Czopek [40] and Engbretson and Hutchinson [41] found no difference in Hb between male and female of the reptiles, but the present study reported a lower percentage of Hb in females of *N. naja*, results that coincide with our previous findings on other reptiles [8,9]. This is may be due to the environment and geographic distribution of the species. There is no sex-based difference in leukocyte count but the lymphocytes are more numerous in male than the female snakes. In addition, the monocyte count is higher in female snakes. The percentages of eosinophils and heterophils are higher in males than the females. In *N. kaouthia*, the basophil count was significantly higher, which depends on species and possibly on season, geographic region, and age of the animal or may be associated with blood parasites or viral infection [5]. Moreover, their work reported that the heterophil count in *Ophiophagus hannah* was significantly higher than that of *N. kaouthia*. As to plasma biochemistry in our study, male and female individuals differed with regard to cholesterol and total protein quantities. Biochemical aspects of the present study indicate higher amounts of proteins and cholesterol in the blood plasma among females than males. Elevated cholesterol levels may be due to a diet normally eaten in the wild.

## Conclusion

In conclusion, hematology and plasma chemistry are highly dependent on physiological processes and the understanding of different factors that govern reference ranges for snakes. More importantly, although hematology is less accurate for snakes than mammals, it is still a useful guideline in the evaluation of health status. Moreover, plasma biochemistry levels in snakes are effective at identifying the changes of visceral organ function prior to a sign of clinical abnormality. However, the small numbers in any specific snake populations limit the interpretation of the results so that further validation is required. As the reproductive programs influence the number of snake species, more research will be necessary to determine the effects of climate, microhabitat, environmental conditions, ambient temperature, and possible seasonal fluctuations on the snake’s hematology and plasma chemistry parameters.

## Competing interests

The authors declare that there are no competing interests.

## Authors’ contributions

SPP contributed to conception and design; acquisition, analysis and interpretation of data; and final approval of the version to be published. SKD contributed to conception and design, revision regarding intellectual content and final approval of the version to be published. AP contributed to conception and design, analysis and interpretation of data, article drafting, revision for regarding intellectual content and final approval of the version to be published.
